# Frequent mutation of hypoxia-related genes in persistent pulmonary hypertension of the newborn

**DOI:** 10.1186/s12931-020-1314-5

**Published:** 2020-02-13

**Authors:** Mingbang Wang, Deyi Zhuang, Mei Mei, Haiyan Ma, Zixiu Li, Fusheng He, Guoqiang Cheng, Guang Lin, Wenhao Zhou

**Affiliations:** 1 Shanghai Key Laboratory of Birth Defects, National Health Commision (NHC) Key Laboratory of Neonatal Diseases, Division of Neonatology, National Center for Children’s Health, Children’s Hospital of Fudan University, Shanghai, 201102 China; 2 Xiamen Key Laboratory of Neonatal Diseases, Neonatal Medical Center, Xiamen Children’s Hospital, Children’s Hospital of Fudan University (Xiamen Branch), Xiamen, 361006 Fujian China; 3 Division of Pulmonology, Children’s Hospital of Fudan University, Shanghai, 201102 China; 4 Zhuhai Maternal and Children’s Hospital, Zhuhai, 519001 Guangdong China; 5 Department of Population and Quantitative Health Sciences, University of Massachusetts Medical School, Worcester, MA 01655 USA; 6 Imunobio, Shenzhen, Guangdong China; 7 Division of Neonatology, Children’s Hospital of Fudan University, Shanghai, 201102 China; 8 Key Laboratory of Birth Defects, Children’s Hospital of Fudan University, Shanghai, 200436 China

**Keywords:** Persistent pulmonary hypertension of the newborn, Whole exome sequencing, Target region sequencing, Hypoxia tolerance

## Abstract

**Aims:**

Persistent pulmonary hypertension of the newborn (PPHN) is characterized by sustained high levels of pulmonary vascular resistance after birth with etiology unclear; Arterial blood oxygen saturation of Tibetan newborns at high latitudes is higher than that of Han newborns at low latitudes, suggesting that genetic adaptation may allow sufficient oxygen to confer Tibetan populations with resistance to pulmonary hypertension; We have previously identified genetic factors related to PPHN through candidate gene sequencing; In this study, we first performed whole exome sequencing in PPHN patients to screen for genetic-related factors.

**Methods and results:**

In this two-phase genetic study, we first sequenced the whole exome of 20 Tibetan PPHN patients and compared it with the published genome sequences of 50 healthy high-altitude Tibetanshypoxia-related genes, a total of 166 PPHN-related variants were found, of which 49% were from 43 hypoxia-related genes; considering many studies have shown that the differences in the genetic background between Tibet and Han are characterized by hypoxia-related genetic polymorphisms, so it is necessary to further verify whether the association between hypoxia-related variants and PPHN is independent of high-altitude life. During the validation phase, 237 hypoxia-related genes were sequenced in another 80 Han PPHN patients living in low altitude areas, including genes at the discovery stage and known hypoxia tolerance, of which 413 variants from 127 of these genes were shown to be significantly associated with PPHN.hypoxia-related genes.

**Conclusions:**

Our results indicates that the association of hypoxia-related genes with PPHN does not depend on high-altitude life, at the same time, 21 rare mutations associated with PPHN were also found, including three rare variants of the tubulin tyrosine ligase-like family member 3 gene (*TTLL3*:p.E317K, *TTLL3*:p.P777S) and the integrin subunit alpha M gene (*ITGAM*:p.E1071D). These novel findings provide important information on the genetic basis of PPHN.

## Introduction

Persistent pulmonary hypertension of the newborn (PPHN) affects 1.9 per thousand live births and is one of the important factors leading to neonatal mortality [1], the main feature of patients with PPHN is that pulmonary vascular resistance cannot be rapidly reduced to increase pulmonary blood flow and oxygen levels and to adapt to the postnatal environment [2]. Although PPHN can be rapidly diagnosed and treated with vasodilators and life support, the mortality rate remains high, at 8–10%. In surviving patients, PPHN can cause neurological damage, cerebral palsy, deafness, blindness, and other complications [3, 4]. Sustained hypoxia resulting from PPHN has been associated with pulmonary vascular dysfunction, alveolar capillary dysplasia, severe lung dysplasia, and progressive lung injury [5]. Epidemiological data also support the association between PPHN and the development of lung disease [5, 6]. Inhaled nitric oxide, a selective pulmonary vasodilator, is widely used in PPHN therapy; however, 30–40% of treated patients, particularly those with pulmonary parenchymal lesions and pulmonary hypoplasia, fail to achieve sustained improvement in oxygenation [7]. These findings suggest that there may be a genetic basis for the disease in some PPHN patients.

Recent studies have shown that genetic factors play an important role in the pathogenesis of pulmonary arterial hypertension (PAH) [8]. However, unlike PAH, PPHN is rarely familial and there has been little research on potential genetic associations [2]. We previously used target region sequencing (TRS) of genes associated with vascular activity in PPHN patients, and we found a significant association between the disease and a variant of the *EDN1* endothelin 1-coding gene [9], recently, we screened clinically relevant mutations in children with PPHN patients through a target panel containing more than 2700 rare disease-related genes [10]. Niermeyer et al. found that the arterial oxygen saturation at birth and in the first 4 months after birth in Tibet (3658 m above sea level) was higher than that in Han newborns at plain, suggesting that genetic adaptation may allow sufficient oxygen to confer resistance to pulmonary hypertension [11]; at the same time, recent genome-wide studies have found that genetic basis of Tibetan population adapts to high-altitude chronic hypoxia, hypoxia-inducible factor pathway gene polymorphisms are significantly different among Tibetans and Han population [12].

In the present study, we sought to extend this study by screening for gene variants associated with PPHN in the high altitudes Tibetan population, which exhibits high adaptability to hypoxia and confers resistance to pulmonary hypertension. We first performed whole exome sequencing (WES) of 20 Tibetan PPHN patients and compared the results with a published dataset of 50 healthy Tibetan individuals to find PPHN-related single nucleotide polymorphisms (SNPs). We then validated in 80 patients from plain PPHN populations to see if the PPHN-related SNPs were independent of high altitudes.

## Materials and methods

### Patients

The high altitudes PPHN patients were recruited from Lhasa People’s Hospital, and the plain PPHN patients were recruited from Children’s Hospital of Fudan University. PPHN was diagnosed by clinical and echocardiographic data, as detailed in our previous study [9]. Informed consent was obtained from a parent, and the study was approved by the ethics committee of the Children’s Hospital of Fudan University (No. 2015–169).

### Whole exome and targeted region sequencing

Genomic DNA was extracted from patient peripheral blood (1–2 ml) samples using a Magbind Blood DNA Kit (CW Biotech, Beijing, China) according to the manufacturer’s instructions and was stored at − 20 °C. WES and TRS were performed as previously described [9, 13]. In brief, whole exomes were captured using SeqCap EZ Exome (44 M) arrays (Roche, Basel, Switzerland). The targeted region was captured using a SeqCap Target Enrichment Kit (Roche) and sequenced using an Illumina HiSeq 2500 System (Illumina, San Diego, CA, USA) at BGI-Shenzhen (BGI, Shenzhen, China).

### Bioinformatics and statistical analysis

#### Discovery stage

For the case–control differential analysis, the raw WES datasets from 20 Tibetan PPHN patients were the cases and published WES datasets from 50 healthy Tibetan individuals [14] were the controls. First, all raw sequencing data were pre-processed using a customized bioinformatics pipeline as described previously [15], and clean reads were generated by removing adapters and filtering out low-quality reads with Trimmomatic [16]. Clean reads were then aligned to the human reference genome (Hg19) using the BWA-MEM algorithm [17] and further visualized using the SplicingViewer software [18], variants were called using GATK [19], and the variants were functionally annotated using ANNOVAR [20]. We then filtered out variants at depths of <20 in the PPHN samples and at depths of <10 in the controls, and Fisher’s exact test was used to determine the difference between PPHN and controls. *P* < 0.001 was considered significant. Genes with PPHN-associated variants were further enriched according to the KEGG pathway database (version 76 [21]). *P* values were determined using Fisher’s exact test, and Q values were determined by false-discovery rate correction of the *P* values. Q < 0.05 was considered significant.

#### Validation stage

To validate the identified PPHN-associated variants in plain population, an additional 80 PPHN patients were sequenced. Variants at depths <20 were filtered out and published 800 healthy controls WES dataset [22] were used as controls for differences analysis using Fisher’s exact test (P value no more than 0.05 were considered significant). The association between gene variants and PPHN was predicted using Phenolyzer [23]. Non-synonymous variants with allelic frequencies of ≤0.01 in the Exome Aggregation Consortium (ExAC) database were considered potential disease-causing variants.

## Results

**Fig. 1:**
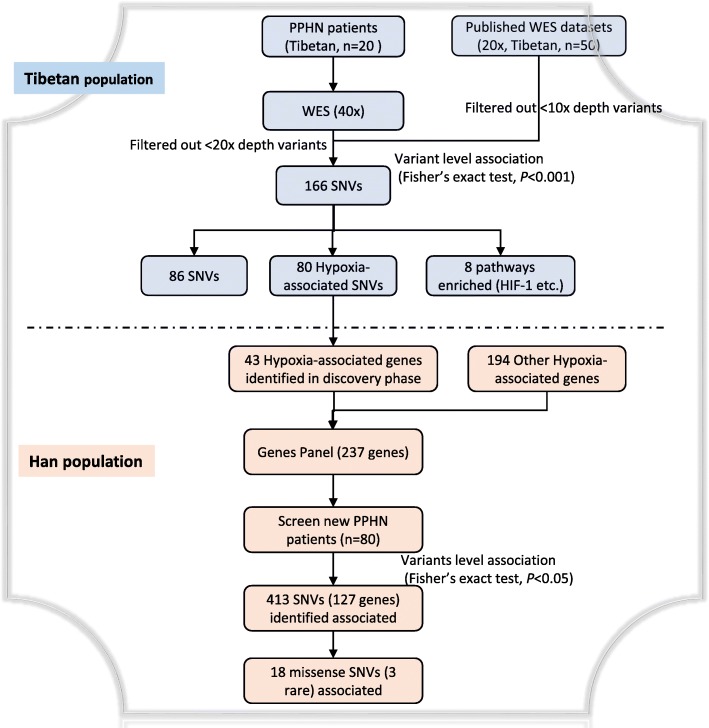
Flowchart of the study design. HIF-1 = hypoxia-inducible factor-1; PPHN = persistent pulmonary hypertension of the newborn; SNVs = single nucleotide variants; WES = whole exome sequencing

A flowchart of the study design is shown in Fig. 1. Clinical information on the study participants is given in Additional file 1: Table S1. In the discovery stage, the average sequencing depth for the 20 Tibetan PPHN patients and 50 Tibetan controls were 40× and 20×, respectively. After strictly filtering out variants with no or low-depth coverage (i.e., < 20 and < 10 for PPHN patients and controls, respectively), a total of 2023 variants were identified (Additional file 1: Table S2) and subjected to case–control differential analysis. Using Fisher’s exact test for single-locus association analysis, a total of 166 significant differences were identified (*P* < 0.001, Additional file 1: Table S3).

In a KEGG pathway analysis, we identified eight pathways enriched in genes with PPHN-associated variants (*Q* < 0.05, Table 1). These were Renal cell carcinoma, Salmonella infection, Pathways in cancer, Graft-versus-host disease, HIF-1 (hypoxia-inducible factor 1) signaling pathway, Bladder cancer, Regulation of actin cytoskeleton, and Gap junction. HIF-1 is a transcription factor that controls the expression of numerous hypoxia-related genes and plays a crucial role in cancer biology [24]. We found that three of the HIF-1 signaling pathway-associated genes; namely, *EGF*, *EP300,* and *IL6,* were also present in the other seven pathways enriched in PPHN-associated variants (Additional file 1: Table S4). Therefore, we turned our focus to genes in the HIF-1 signaling pathway. Interestingly, 49% (80/166) of the PPHN-associated variants were genes regulated by hypoxia or related to hypoxia tolerance (Additional file 1: Table S5); we refer to these as hypoxia-related genes.

**Table 1:** Pathways enriched in PPHN-Associated gene variants identified in the tibetan population

Pathway name	Pathway ID	PPHN genes (114)	All genes (17251)	*P* value	Q value
Renal cell carcinoma	ko05211	7	101	4.71E-06	0.001
Salmonella infection	ko05132	10	317	4.79E-05	0.005
Pathways in cancer	ko05200	14	644	8.85E-05	0.006
Graft-versus-host disease	ko05332	5	74	0.0001293	0.006
HIF-1 signaling pathway	ko04066	7	185	0.0002281	0.009
Bladder cancer	ko05219	4	61	0.0007062	0.023
Regulation of actin cytoskeleton	ko04810	11	588	0.0017709	0.050
Gap junction	ko04540	5	135	0.0020232	0.050

Definition of abbreviations: *HIF-1* Hypoxia-inducible factor-1, *PPHN* Persistent pulmonary hypertension of the newborn

To verify whether the correlation between hypoxia-related associated genes and PPHN is independent of high altitude, we first conducted a systematic literature search and identified 246 hypoxia-related genes (Additional file 1: Table S5), and then designed a capture chip that captures the exon regions of 246 hypoxia-related genes, finally, the targeted region was sequenced in 80 plain PPHN patients with an average depth of 100x. As in the discovery stage, we use strict filtering standards to remove variants with no or low-depth coverage in the controls, and identified a total of 413 variants significantly associated with PPHN (*P* < 0.05, Additional file 1: Table S6) in 127 of the 246 hypoxia-related genes. Of these 413, only 5% (21) were population-specific variants (*P* < 0.05, Additional file 1: Table S7) and were not considered rare (minor allele frequency [MAF] > 0.05 in the ExAC database). Most of these variants (20/21) were located in the intronic or exonic regions and did not result in amino acid changes. One missense variant was associated with the *FANCA* (Fanconi anemia complementation group A) gene.

Eighteen of the PPHN-associated variants consisted of non-synonymous mutations in 14 genes; *ANP32D, C12orf54, DR1, DUOXA1, FANCA, ITGAM, MBL2, MDH1B, PFKM, PLAU, PTX3, SIPA1L2, TMEM206,* and *TTLL3* (Table 2). Most of these variants (15/18) were not rare (MAF > 0.01) in ExAC. The three rare (MAF < 0.01) variants were two missense mutations in *TTLL3* (tubulin tyrosine ligase-like 3), p.E317K and p.P777S, and one missense mutation in *ITGAM* (integrin subunit alpha M), p.E1071D. Both *TTLL3* and *ITGAM* were predicted to be associated with the PPHN phenotype using Phenolyzer (*P* = 0.002589 and *P* = 0.03325, respectively).

**Table 2:** PPHN-Associated missense variants identified in the han population

Chr	Pos	Ref	Alt	Variants	Gene	PPHN (MAF)	Ctrl (MAF)	*P* value	OR	MAF^a^
chr12	48,866,585	A	C	p.L46F	*ANP32D*	0.604	0.687	0.0487	0.69	0.187
chr12	48,888,594	C	T	p.P86S	*C12orf54*	0.188	0.12	0.0265	1.70	0.372
chr1	93,826,178	A	T	p.E171D	*DR1*	0.052	0.016	0.0121	3.31	0.092
chr15	45,409,732	C	G	p.R433P	*DUOXA1*	0.071	0.033	0.0365	2.28	0.083
chr16	89,857,935	G	A	p.A412V	*FANCA*	0.156	0.29	0.0005	0.45	0.065
chr16	89,805,914	T	C	p.T1328A	*FANCA*	0.156	0.274	0.0017	0.49	0.031
chr16	89,839,766	G	C	p.P643A	*FANCA*	0.182	0.297	0.0032	0.53	0.025
chr16	89,836,323	C	T	p.G809D	*FANCA*	0.968	0.99	0.0443	0.30	0.467
chr16	31,341,863	G	C	p.E477D	*ITGAM*	0.026	0.005	0.027	5.29	0.001
chr10	54,531,235	C	T	p.G54D	*MBL2*	0.13	0.209	0.0262	0.57	0.139
chr2	207,603,221	T	C	p.T515A	*MDH1B*	0.052	0.021	0.0475	2.52	0.087
chr12	48,501,161	A	T	p.H2L	*PFKM*	0.532	0.621	0.0473	0.70	0.266
chr10	75,673,101	T	C	p.L105P	*PLAU*	0.558	0.682	0.0038	0.59	0.755
chr3	157,155,314	C	A	p.A48D	*PTX3*	0.857	0.775	0.0234	1.74	0.625
chr1	232,539,219	C	T	p.G695S	*SIPA1L2*	0.442	0.544	0.0218	0.66	0.065
chr1	212,587,320	T	C	p.N47D	*TMEM206*	0.162	0.088	0.0076	2.02	0.035
chr3	9,876,568	C	T	p.P777S	*TTLL3*	0.026	0.005	0.027	5.29	0.001
chr3	9,860,595	G	A	p.E317K	*TTLL3*	0.006	0.038	0.0463	0.17	0.001

^a^Minor allele frequency (MAF) according to the Exome Aggregation Consortium database. Definition of abbreviations: *Alt* Alteration, *Chr* Chromosome, *Ctrl* Control, *Pos* Position, *OR* Odds ratio, *PPHN* Persistent pulmonary hypertension of the newborn, *Ref* Reference

## Discussion

In this two-stage study, we first compared WES data from 20 Tibetan PPHN patients and 50 healthy Tibetan controls living at high altitudes and identified 166 PPHN-associated variants, 49% of which were derived from 43 hypoxia-related genes; considering many studies have shown that the differences in the genetic background between Tibet and Han are characterized by hypoxia-related genetic polymorphisms [12]; to further verify whether the association of hypoxia-associated variants with PPHN is independent of high altitude life, we performed a targeted validation study of hypoxia-related genes in an additional 80 Han PPHN patients living at low altitudes. Finally, we discovered 413 PPHN-associated variants from 127 hypoxia-related genes in Han population.

Significant changes in blood coagulation at high altitudes may predispose individuals to pulmonary hypertension [25, 26]. Negi et al. [27] conducted a cross-sectional survey of permanent inhabitants in the Spiti Valley at an altitude of 3000 to 4200 m, although no significant correlation was found between altitude and pulmonary hypertension. However, it is still found that the prevalence rate is increasing with the increase of altitude; the data published to date have not been identified in plateau populations as genetic susceptibility to individual pulmonary hypertension [28, 29]. Our results indicates that the association of hypoxia-related genes with PPHN does not depend on high-altitude life, at the same time, some rare mutations associated with PPHN were also found.

Two rare missense mutations identified in *TTLL3* (p.E317K and p.P777S) were associated with PPHN. TTLL3 catalyzes the ATP-dependent restoration of tyrosine to the C-terminus of α-tubulin, which polymerizes into microtubules [30, 31]. Microtubule proliferation has been found in the right ventricular myocytes of rats with monocrotaline-induced pulmonary hypertension, accompanied by upregulated expression of α- and β-tubulin [32]. The detyrosination/tyrosination cycle of tubulin is important for regulating the mechanical stabilization of long-lived microtubules in cells, and abnormalities in this cycle are involved in the development of hypertension [33]. A recent study found that TTLL3 can also ligate various unnatural amino acids to the C-terminus of tubulin [34]. The crystal structure of TTLL3 has recently been solved and suggests that TTLL3 can ligate glycine and glutamate to the tubulin C-terminus, a process crucial for the biogenesis and stability of microtubules in cilia, flagella, spindles, neuronal processes, and platelets [35, 36]. Glutamylation, the most prevalent post-translational modification of tubulin, stabilizes microtubules and regulates the recruitment and activity of microtubule-interacting proteins [37]. Valenstein et al. found that glutamylation was a major regulator of hereditary spastic paraplegia [38]. Consistent with the potential importance of microtubule-associated proteins in PPHN, we found significant enrichment of genes associated with regulation of the actin cytoskeleton among the 166 PPHN-associated variants identified in the Tibetan population. These included *TTLL3, EGF*, *GOLGA1*, *KRAS*, *IQGAP1*, *LRRFIP2*, *RB1CC1*, *TEX14*, *GCOM1*, *ARPC4-TTLL3,* and *MYZAP*. Interestingly, Fediuk et al. found that the eicosanoid thromboxane can induce actin polymerization in hypoxic neonatal pulmonary arterial myocytes [39, 40].

Rare missense mutation identified in in *ITGAM* (p.E1071D) was associated with PPHN, *ITGAM* encodes the integrin alpha M chain, also known as Mac-1, CD11b/CD18, or CR3A. Integrins play important roles in cell–cell adhesion by mediating transmembrane connections to the cytoskeleton and activating intracellular signaling pathways. A previous study showed that integrins are differentially regulated in pulmonary artery smooth muscle cells during pulmonary hypertension [41]. In addition, pulmonary artery endothelial cells from patients with idiopathic pulmonary hypertension show decreased adhesion to laminin, and loss of interaction between α3 integrins and the tumor promoter APC (adenomatous poliposis coli) promotes endothelial apoptosis in mice and humans [42]. Furthermore, a recent study showed that induced pluripotent stem cell-derived endothelial cells from a patient with familial PAH showed reduced adhesion compared with control cells [43]. The missense mutation identified in the present study, p.E1071D, lies in the C-terminal domain of *ITGAM*, which may be responsible for recognizing short peptide motifs [44] that are involved in integrin–extracellular matrix interactions. This suggests a potential mechanism by which aberrant behavior of the ITGAM variant may contribute to PPHN.

Nevertheless, it is unclear how this integrin may be involved in PAH or PPHN or whether the *ITGAM* mutation is protective or a risk factor. Jiang et al. found that platelet-mediated mesenchymal stem cell homing to the lung can reduce monocrotaline-induced rat pulmonary hypertension [45]. A recent study showed that ITGAM can regulate thrombosis via interaction with platelet GPIb [46], suggesting that it may play a protective function against thrombosis. However, upregulation of integrins may also be associated with the onset of PPHN, as previous studies have shown that integrins αvβ6 and αvβ8 are upregulated in bronchial epithelial cells from patients with systemic sclerosis, a disease often associated with PAH [47]. Welschoff et al. found that the tripeptide Arg-Gly-Asp, which inhibits the adhesion of several integrins, can induce relaxation of pulmonary arteries and reduce pulmonary arterial pressure [48].

PPHN is not a single disease, but a clinical syndrome caused by multiple factors, that may present with different clinical conditions requiring different treatments and with variable outcomes. The PPHN related genetic variants we described could improve the understanding of the pathogenesis of PPHN and, consequently, increase the spectrum of available treatments by targeting hypoxia related gene pathways [49, 50] . Finally our paper may also suggest the feasibility of whole-genome sequencing for the screening of clinically relevant mutations, associated with PPHN.

This study has some innovations. To the best of our knowledge, we have performed exome screening for the first time in PPHN patients; we found that hypoxia-related genes are associated with PPHN and are not dependent on high altitudes. It provides a genetic basis for the pathogenesis of PPHN and also provides a target for our PPHN genetic screening in newborns. There are some limitations to our stud*y.* The published Tibetan WES datasets used as controls were of low coverage, and the rigorous filtering may have eliminated Tibetan-specific variants associated with PPHN. Although we have identified three rare missense variants associated with PPHN, further functional studies will be necessary to understand how they contribute to the pathogenesis of PPHN.

## Conclusions

We conducted a two-stage genetic study of 100 PPHN patients and identified several hypoxia-associated gene variants that may be associated with PPHN, and the association of hypoxia-associated gene mutations with PPHN does not depend on high altitude life.

## Supplementary information

**Additional file 1: Table S1.** Clinical information on study participants. **Table S2.** List of 2023 variants used for case–control analysis in Tibetan population. **Table S3.** List of 166 gene variants significantly associated with PPHN (*P* < 0.001) identified in the Tibetan population. **Table S4.** List of pathways enriched in the 166 variants identified in the Tibetan population. **Table S5.** List of 237 hypoxia-related genes studied in the Han population. **Table S6.** List of 413 variants significantly associated with PPHN (*P* < 0.05) identified in the Han population. **Table S7.** List of 21 population-specific variants significantly associated with PPHN (*P* < 0.05).

## Data Availability

The datasets for this study can be found in the supplemental material, and the raw data can be obtained from the corresponding author under request.

## Abbreviations

ExAC: Exome Aggregation Consortium
KEGG: Kyoto Encyclopedia of Genes and Genomes
PAH: Pulmonary arterial hypertension
PPHN: Persistent pulmonary hypertension of the newborn
TRS: Target region sequencing
WES: Whole exome sequencing
